# Association News

**Published:** 1901-07

**Authors:** 


Secretary.

---

### ASSOCIATION NEWS.

The veterinarians employed by the Bureau of Animal Industry at Kansas City have recently formed an organization for mutual help and improvement. Regular meetings will be held on the first Monday evening of each month.

The following gentlemen are the officers for the current quarter: Dr. S. E. Bennett, President; Dr. H. H. George, First Vice-President; Dr. W. U. Neil, Second Vice-President; Dr. J. S. Grove, Secretary and Treasurer.

